# Microevolution and Patterns of Transmission of *Shigella sonnei* within Cyclic Outbreaks Shigellosis, Israel

**DOI:** 10.3201/eid2407.171313

**Published:** 2018-07

**Authors:** Adi Behar, Kate Susan Baker, Ravit Bassal, Analia Ezernitchi, Lea Valinsky, Nicholas R. Thomson, Daniel Cohen

**Affiliations:** Tel Aviv University, Tel Aviv, Israel (A. Behar, D. Cohen); Kimron Veterinary Institute, Beit Dagan, Israel (A. Behar);; The Wellcome Trust Sanger Institute, Hinxton, Cambridge, UK (K.S. Baker, N.R. Thomson); University of Liverpool, Liverpool, UK (K.S. Baker);; Israel Center for Disease Control, Jerusalem, Israel (R. Bassal);; Central Laboratories, Ministry of Health, Jerusalem (A. Ezernitchi, L. Valinsky)

**Keywords:** Whole-genome sequencing, *Shigella* *sonnei*, *flexneri*, shigellosis, microevolution, ultraorthodox Jews, secular Jews, Israeli Arabs, person-to-person transmission, overcrowding, tetracycline, children, migration, Bedouin, Beer Sheva, Mevaseret, Zion, Israel

## Abstract

Whole-genome sequencing unveiled host and environment-related insights to *Shigella sonnei* transmission within cyclic epidemics during 2000–2012 in Israel. The Israeli reservoir contains isolates belonging to *S. sonnei* lineage III but of different origin, shows loss of tetracycline resistance genes, and little genetic variation within the O antigen: highly relevant for *Shigella* vaccine development.

Shigellosis is common all over the world and is hyperendemic to developing countries where children with the disease have an increased risk for persistent diarrhea, arrested growth, and death (*1*–*3*). The annual incident cases of shigellosis are estimated at ≈190 million in developing countries, where *Shigella flexneri* is the most common cause of shigellosis, and ≈1 million in industrialized countries, where *S. sonnei* predominates (*4*–*7*).

## The Study

Despite the improved socioeconomic conditions, Israel has remained an area where shigellosis is highly endemic, reporting an annual incidence rate of culture-proven shigellosis of ≈97 cases per 100,000 population. Cyclic outbreaks during 2000–2012 occur every 2 years; *S. sonnei* is the pathogen for >85% of the cases. It has been shown that the ultraorthodox Jewish communities, which are overcrowded and have a high number of children <5 years of age, were the epicenter of these epidemics during the past 15 years (*5*). We used whole-genome sequencing (WGS) to provide a high-resolution view to better understand the local microevolution and patterns of *S. sonnei* transmission within the cyclic outbreaks in Israel.

**Figure 1:**
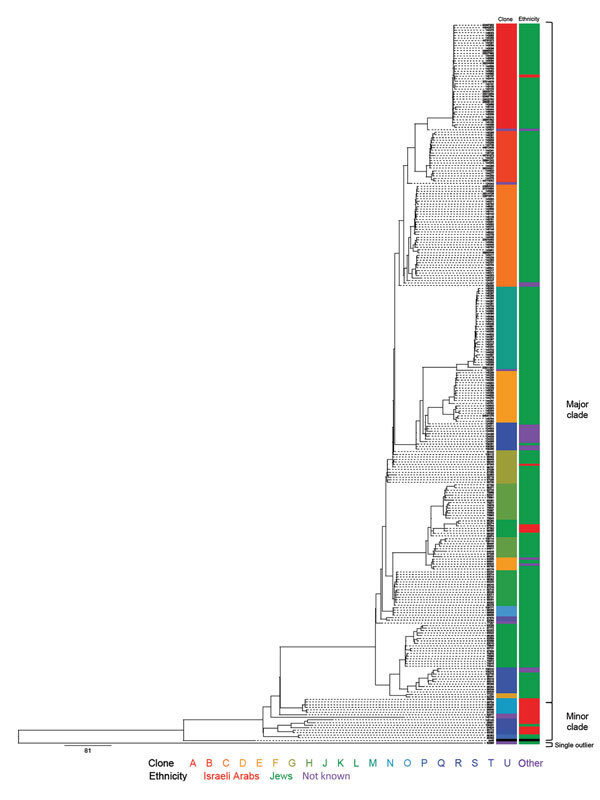
Whole-genome sequencing and phylogenetic analysis of 281 *Shigella sonnei* isolated in Israel, 2000–2012. Scale bar indicates single-nucleotide polymorphisms.

A total of 281 *S. sonnei* isolates were subject to WGS (Figure 1; Technical Appendix Table 1). We collected data from isolates during the epidemic years 2000, 2002, 2004, 2006, 2008, and 2012, and the nonepidemic years 2001 and 2003. All isolates were from children of various sanitary, socioeconomic, cultural, and ethnic backgrounds: ultraorthodox Jews, secular Jews, and Israeli Arabs. The ultraorthodox Jews represent ≈11% of the total population of Israel. This population group resides in towns or neighborhoods separated from the secular Jewish population (*8*) and also in mixed ones. The Israeli Arabs, who are estimated to account for 20% of the total population, reside mostly in rural areas and in towns or neighborhoods separated from the Jewish population; but they also live in towns inhabited by both Jews and Arabs (*8*). Of the 281 isolates, 263 (93.5%) were collected from Jewish children (mainly from ultraorthodox communities) and 18 (6.4%) isolates were from Israeli Arab children (mainly Bedouins living in southern Israel).

The WGS analysis showed that the clones within the Israeli reservoir formed 2 distinct subclades: a major subclade (subclade I) containing ≈94% of the Israeli collection, which is more prevalent among Jewish children (92% originated from Jewish children); and a minor subclade (subclade II) containing ≈5.7% of the Israeli collection, which is more prevalent among Israeli Arab children (82%). Only 1 isolate (≈0.3%) did not cluster with any of the Israeli isolates (Figure 1).

A comparison to global analyses (*9*) suggests that even though both subclades belong to *S. sonnei* lineage III, they are of different origins. Subclade II clones were more closely related to isolates that originated in Egypt and Iran than to the Israeli subclade I clones that seem to be endemic and have a distinctive recombination site, as previously described for 1 sequenced isolate from a patient in Israel in 2003 (*9*; Technical Appendix Table 2). They were also found to distinguish *S. sonnei* among Jewish Orthodox communities of various countries (*10*). Nine of 13 Israeli Arab strains in clade II were isolated from Bedouins living in the vicinity of the Egyptian border. The frequent migration over the Israel–Egypt border of Bedouins often belonging to the same tribe could explain the possible importation of subclade II *S. sonnei* from Egypt and/or through Egypt, similar to the recent transborder silent spread of poliovirus type 1, another fecal–orally transmitted enteropathogen in southern Israel (*11*). Our results also indicate that in general, isolates from Israeli Arab children who reside in mixed settlements and in close proximity to Jewish children commonly have positive test results for *Shigella* strains in clade 1. Only 5 (1.9%) isolates in clade I originated from Israeli Arabs (Figure 1). Of note, 4 of the 5 isolates were obtained from samples from Arab children residing in Beer Sheva (3 isolates) and Mevaseret Zion (1 isolate), cities inhabited by both Jews and Arabs. Consequently, it appears that a combination of both biogeography and ethnicity forming microhabitats for *S. sonnei* clone circulation shapes the differences observed between Jewish and Israeli Arab children.

**Figure 2:**
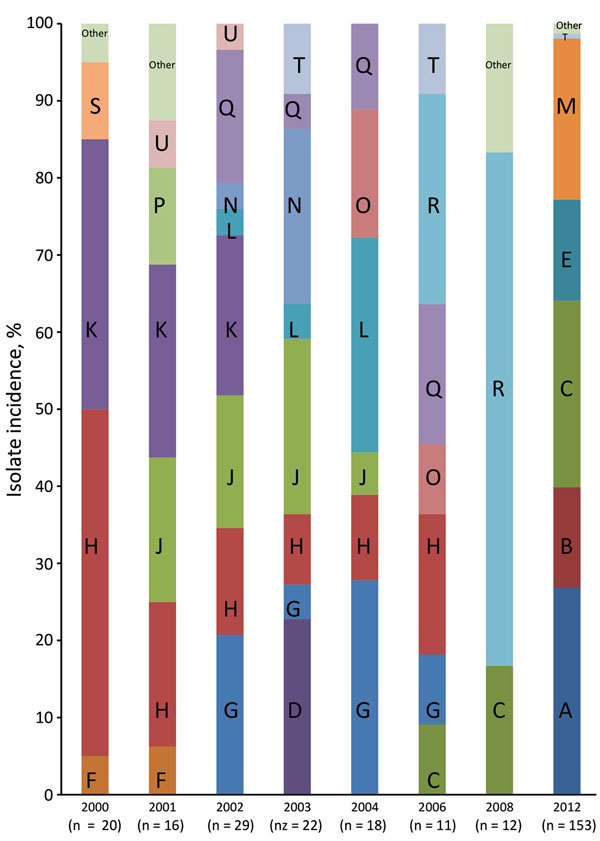
The relative distribution of the various whole-genome sequencing defined *Shigella sonnei* clones per year of isolation, 2000–2012.

Each subclade could be further subdivided into clonal groups consisting of clusters of isolates with <30 chromosomal single-nucleotide polymorphism (SNP) differences from the nearest neighboring cluster. We defined a total of 20 unique and distinct *S. sonnei* endemic clones circulating in the Israeli population (Figure 2, panels A–U). The majority of the clones (≈69%; Figure 2) can be found throughout the years regardless of the shigellosis outbreaks that occurred in Israel every 2 years during 2000–2012, suggesting some mechanism of persistence (*5*). Contrary to our hypothesis, neither the establishment and dynamics of persistent or dominant clones could explain the Israeli cyclic outbreaks. Moreover, we found no specific genetic attributes that could distinguish them from other clones. Therefore, we postulate that the cyclic peaks of morbidity rates associated with *S. sonnei* are the result of changes in the level of natural immunity, as was shown by several observational studies (*5*,*12*,*13*). An outbreak of shigellosis occurring among children 0–4 years of age will lead to an increase in the level of natural immunity to the homologous *Shigella* organism (*S. sonnei*), which will also provide the level of herd immunity sufficient to prevent the onset of a new epidemic. After 1 or 2 years, declining levels of antibodies together with the intake of a new cohort of naive newborns will lead to a decrease in the level of herd immunity below a critical level. High and continuous exposure to a variety of circulating *S. sonnei* clones in children 0–4 years of age who live in crowded conditions will lead to the renewal of the epidemic transmission of these clones (*5*).

## Conclusions

Although we excluded all *S. sonnei* plasmids from the phylogenetic analysis, plasmid reads were mapped and the assembled sequences compared with the reference plasmid sequences. Our data suggest that plasmid spA is undergoing degradation as a result of the loss of tetracycline resistance genes over time. This finding is consistent with the results of Holt et al. for the Middle East (III) clade (*9*) and with laboratory examination showing that the *S. sonnei* Israeli reservoir is becoming less resistant to tetracycline (*5*) (p-value for linear trend <0.01) (Technical Appendix Table 3).

Although notoriously unstable when *S. sonnei* is grown on laboratory media, invasive plasmid pINVB was present in ≈58% of our isolate sequences. Our results demonstrate that *S. sonnei* O antigen encoded on this plasmid is well-conserved within the *S. sonnei* Israeli reservoir. No SNPs were detected in genes that belong to the O antigen gene cluster in ≈97% of the plasmids, and pINVB seems to be under very little immune selection as has been also shown in other studies (*9*,*14*). We identified a single SNP leading to a nonsynonymous substitution, in gene *wbgW* within the O antigen gene cluster that was shared by only 4 (2.4%) isolate plasmids. We also identified in 1 (≈0.6%) isolate 1 SNP, a nonsynonymous change in gene *wbgY.* To date, *Shigella* vaccine development has mainly focused on serotype-targeted vaccines that are based on *Shigella* O antigen (*15*). Thus, our findings may have implications for public health as the need for a safe and effective *Shigella* vaccine becomes more pressing (*15*).

Technical Appendix Part 1) Methodology used for whole-genome sequencing and SNP–based analysis. Part 2) Israeli sub-clade positions within the global S. sonnei phylogeny.
