# Supplementary material for: Microevolution and Patterns of Transmission of Shigella sonnei within Cyclic Outbreaks Shigellosis, Israel
Source: Emerg Infect Dis. 2018 Jul;24(7):1335–9. doi: 10.3201/eid2407.171313 (PMC6038740; doi:10.3201/eid2407.171313)
Supplement: Technical Appendix — Part 1) Methodology used for whole-genome sequencing and SNP–based analysis. Part 2) Israeli sub-clade positions within the global S. sonnei phylogeny. [file 17-1313-Techapp-s1.pdf]

Article DOI: https://doi.org/10.3201/eid2407.171313

Microevolution and Patterns of Transmission of *Shigella sonnei* within Cyclic Outbreaks of Shigellosis, Israel

Technical Appendix Part 1: The Methodology used in this Study for Whole-genome Sequencing and SNP–based Analysis

For whole genome sequencing, bacterial DNA from *S. sonnei* isolates was extracted from colonies by using Wizard Genomic DNA Purification Kit (Promega, Madison, WI, USA) according to the manufacturer's protocol. Index-tagged paired-end Illumina sequencing libraries were prepared, as previously described (*1*) and were sequenced on the Illumina Genome Analyzer GAII according to the manufacturer's protocols to generate tagged paired-end reads. Reads from each isolate were mapped to the *S. sonnei* reference genome (strain Ss046 chromosome: NC_007384; strain Ss046 plasmids: NC_007385, NC_009347, NC_009346 and NC_009345; plasmid pEG356: NC_013727) by using Bowtie (*2*) to produce a binary alignment map (BAM). SAMtools34 (*3*) was used to create a variant call format (VCF) file from each of the BAMs, which was further parsed to extract only single nucleotide polymorphism (SNP) positions which were of high quality in all genomes. Regions of unexpectedly high SNP density that might have been introduced by mobile element movement or recombination, were removed from the alignment by using gubbins (*4*), and a maximum-likelihood phylogeny was derived using RAxML (*5*). Gene content were examined manually using the comparative genomic approaches in Artemis and ACT (*6*). Welcome Trust Sanger Institute sequence data are available in the Short Read Archive under the accession numbers provided in Technical Appendix Table 1.

Technical Appendix References

1. Harris SR, Feil EJ, Holden MT, Quail MA, Nickerson EK, Chantratita N, et al. Evolution of MRSA during hospital transmission and intercontinental spread. Science. 2010;327:469–74. PubMed http://dx.doi.org/10.1126/science.1182395

2. Langmead B, Schatz MC, Lin J, Pop M, Salzberg SL. Searching for SNPs with cloud computing. Genome Biol. 2009;10:R134. PubMed http://dx.doi.org/10.1186/gb-2009-10-11-r134

3. Li H, Handsaker B, Wysoker A, Fennell T, Ruan J, Homer N, et al.; 1000 Genome Project Data Processing Subgroup. The sequence alignment/map format and SAMtools. Bioinformatics. 2009;25:2078–9. PubMed http://dx.doi.org/10.1093/bioinformatics/btp352

4. Croucher NJ, Page AJ, Connor TR, Delaney AJ, Keane JA, Bentley SD, et al. Rapid phylogenetic analysis of large samples of recombinant bacterial whole genome sequences using Gubbins. Nucleic Acids Res. 2015;43:e15. PubMed http://dx.doi.org/10.1093/nar/gku1196

5. Stamatakis A. RAxML-VI-HPC: maximum likelihood-based phylogenetic analyses with thousands of taxa and mixed models. Bioinformatics. 2006;22:2688–90. PubMed http://dx.doi.org/10.1093/bioinformatics/btl446

6. Carver T, Harris SR, Berriman M, Parkhill J, McQuillan JA. Artemis: an integrated platform for visualization and analysis of high-throughput sequence-based experimental data. Bioinformatics. 2012;28:464–9. PubMed http://dx.doi.org/10.1093/bioinformatics/btr703

7. Holt KE, Baker S, Weill FX, Holmes EC, Kitchen A, Yu J, et al. *Shigella sonnei* genome sequencing and phylogenetic analysis indicate recent global dissemination from Europe. Nat Genet. 2012;44:1056–9. PubMed http://dx.doi.org/10.1038/ng.2369

Technical Appendix Part 2: Israeli sub-clade positions within the global *S. sonnei* phylogeny determined by Holt et al. (*7*)

Technical Appendix Table 1. Accession numbers for the 281 isolates included in this study.

Identification number	Accession number
22672674	ERR190914
26986104	ERR190906
29022762	ERR190916
35527695	ERR190911
36230921	ERR190913
36234868	ERR190907
36303731	ERR190915
36314706	ERR190910
36314959	ERR190908
36860531	ERR190909
36860548	ERR190912
36861682	ERR190917
36991242	ERR190905
44773052	ERR211146
90931857	ERR211147
37975463	ERR211148
94081275	ERR211149

Identification number	Accession number
44672409	ERR211150
44964030	ERR211151
44942670	ERR211152
37514976	ERR211153
37370664	ERR211154
92497764	ERR211155
44781973	ERR211156
92497962	ERR211157
44781873	ERR211158
102735	ERR190851
109112	ERR190852
109113	ERR190853
109114	ERR190854
109171	ERR190855
109201	ERR190856
109251	ERR190857
109275	ERR190858
109280	ERR190859
109295	ERR190860
109305	ERR190861
109310	ERR190862
124327	ERR190893
124332	ERR190894
124342	ERR190895
124357	ERR190896
124496	ERR190902
124511	ERR190903
124521	ERR190904
124528	ERR190899
124553	ERR190901
124558	ERR190900
124561	ERR190897
124566	ERR190898
75544	ERR190745
75637	ERR190746
75643	ERR190747
75645	ERR190748
75659	ERR190749
75668	ERR190750
75672	ERR190751
75676	ERR190752
75677	ERR190753
75679	ERR190754
75684	ERR190755
75686	ERR190756
75691	ERR190757
75693	ERR190758
75887	ERR190759
75915	ERR190760
77231	ERR190761
77257	ERR190762
80080	ERR190763
80113	ERR190764
82064	ERR190767
82205	ERR190768
82541	ERR190769
82721	ERR190770
82723	ERR190771
82817	ERR190772
82896	ERR190773
83010	ERR190774
83011	ERR190775
83361	ERR190776
83588	ERR190777
83589	ERR190778
85001	ERR190779
85002	ERR190780
85359	ERR190781
85362	ERR190782

Identification number	Accession number
85516	ERR190783
85674	ERR190784
85715	ERR190785
85719	ERR190786
85720	ERR190787
85721	ERR190788
86255	ERR190789
86278	ERR190790
86283	ERR190791
86298	ERR190792
86318	ERR190793
86323	ERR190794
86328	ERR190795
87006	ERR190796
87016	ERR190797
87026	ERR190798
87036	ERR190799
87065	ERR190800
87669	ERR190801
87670	ERR190802
87772	ERR190803
87780	ERR190804
88371	ERR190805
89349	ERR190806
92353	ERR190807
92360	ERR190808
93028	ERR190809
93522	ERR190810
94160	ERR190811
94817	ERR190812
94818	ERR190813
94824	ERR190814
94825	ERR190815
95013	ERR190816
95014	ERR190817
95015	ERR190818
95233	ERR190819
95234	ERR190820
95522	ERR190821
95529	ERR190822
95641	ERR190823
95776	ERR190824
95780	ERR190825
96428	ERR190826
96548	ERR190827
96754	ERR190828
96801	ERR190829
96851	ERR190830
96924	ERR190831
97043	ERR190832
97044	ERR190833
97382	ERR190834
98274	ERR190835
98284	ERR190836
98904	ERR190837
98909	ERR190838
99020	ERR190839
99040	ERR190840
99362	ERR190841
99366	ERR190842
99382	ERR190843
99383	ERR190844
99441	ERR190845
99442	ERR190846
99443	ERR190847
99444	ERR190848
99608	ERR190849
99767	ERR190850
143038	ERR319184

Identification number	Accession number
143094	ERR319185
143098	ERR319186
143099	ERR319187
143100	ERR319188
143101	ERR319189
143102	ERR319190
143105	ERR319191
143204	ERR319192
143205	ERR319193
143207	ERR319194
143208	ERR319195
143209	ERR319196
143267	ERR319197
143269	ERR319198
143411	ERR319199
143413	ERR319200
143414	ERR319201
143415	ERR319202
143558	ERR319203
143560	ERR319204
143561	ERR319205
143562	ERR319206
143563	ERR319207
143564	ERR319208
143701	ERR319209
143702	ERR319210
143703	ERR319211
143704	ERR319212
143778	ERR319213
143779	ERR319214
143780	ERR319215
143781	ERR319216
143953	ERR319217
143969	ERR319218
143970	ERR319219
143981	ERR319220
143982	ERR319221
143983	ERR319222
143985	ERR319223
143986	ERR319224
144205	ERR319225
144217	ERR319226
144226	ERR319227
144237	ERR319228
144239	ERR319229
144241	ERR319230
144256	ERR319231
144259	ERR319232
144260	ERR319233
144263	ERR319234
144267	ERR319235
144277	ERR319236
144279	ERR319237
144280	ERR319238
144281	ERR319239
144286	ERR319240
144287	ERR319241
144288	ERR319242
144290	ERR319243
144291	ERR319244
144303	ERR319245
144353	ERR319246
144357	ERR319247
144358	ERR319248
144366	ERR319249
144367	ERR319250
144368	ERR319251
144371	ERR319252
144374	ERR319253

Identification number	Accession number
144375	ERR319254
144376	ERR319255
144444	ERR319256
144445	ERR319257
144446	ERR319258
144555	ERR319259
144587	ERR319260
144630	ERR319261
144632	ERR319262
144635	ERR319263
144636	ERR319264
144642	ERR319265
144668	ERR319266
144669	ERR319267
144748	ERR319268
144885	ERR319269
144890	ERR319270
144891	ERR319271
145087	ERR319272
145088	ERR319273
145089	ERR319274
145090	ERR319275
145091	ERR319276
145137	ERR319277
145138	ERR319278
145139	ERR319279
145140	ERR319280
145141	ERR319281
145142	ERR319282
145143	ERR319283
145145	ERR319284
145146	ERR319285
145295	ERR319286
145296	ERR319287
145297	ERR319288
145298	ERR319289
145299	ERR319290
145304	ERR319291
145308	ERR319292
145309	ERR319293
145310	ERR319294
145311	ERR319295
145312	ERR319296
145550	ERR319297
145560	ERR319298
145583	ERR319299
145641	ERR319300
145698	ERR319301
145781	ERR319302
145782	ERR319303
145783	ERR319304
145784	ERR319305
145999	ERR319306
146000	ERR319307
146001	ERR319308
146002	ERR319309
146003	ERR319310

Technical Appendix Table 2. Similarity of the Israeli minor sub-clade isolates with global *S. sonnei* mapping by Holt et al. (*7*)

Name	Clone	Ethnicity	Isolation date	Similarity with isolates from:
80080	S	Israeli Arabs	2000	Egypt
80113	S	Israeli Arabs	2000	Egypt
85359	P	Jews	2001	Korea
85362	P	Jews	2001	Korea
82817	Other	Israeli Arabs	2001	UK
94160	N	Israeli Arabs	2002	Iran
96851	N	Israeli Arabs	2003	Iran
96924	N	Israeli Arabs	2003	Iran
96428	N	Israeli Arabs	2003	Iran
96548	N	Israeli Arabs	2003	Iran
96754	N	Israeli Arabs	2003	Iran
94824	T	Israeli Arabs	2003	Egypt
96801	T	Israeli Arabs	2003	Egypt
109201	T	Jews	2006	Egypt
145143	T	Israeli Arabs	2012	Egypt
145140	Other	Israeli Arabs	2012	UK
One isolate that did not clade with any of the Israeli strains (belong to Global II)				
82896	Other	Jews	2001	Brazil, Senegal

Technical Appendix Table 3. Resistance of *S. sonnei* isolates to tetracycline (2000–2012)

Year	Total number of isolates	Tetracycline-resistant isolates*	%
2000	20	20	100
2001	16	10	62.5
2002	29	8	27.5
2003	22	6	27
2004	18	3	17
2006	11	0	0
2008	12	0	0
2012	153	29	19

p for linear trend <0.01*
